# Local γδ T cells: translating promise to practice in cancer immunotherapy

**DOI:** 10.1038/s41416-023-02303-0

**Published:** 2023-06-13

**Authors:** Iva Zlatareva, Yin Wu

**Affiliations:** 1 Peter Gorer Department of Immunobiology, King’s College London, London, SE1 9RT UK; 2 Centre for Inflammation Biology and Cancer Immunology, King’s College London, London, SE1 9RT UK; 3 Department of Medical Oncology, Guy’s Hospital, London, SE1 9RT UK

**Keywords:** Tumour immunology, Tumour immunology

## Abstract

Rapid bench-to-bedside translation of basic immunology to cancer immunotherapy has revolutionised the clinical practice of oncology over the last decade. Immune checkpoint inhibitors targeting αβ T cells now offer durable remissions and even cures for some patients with hitherto treatment-refractory metastatic cancers. Unfortunately, these treatments only benefit a minority of patients and efforts to improve efficacy through combination therapies utilising αβ T cells have seen diminishing returns. Alongside αβ T cells and B cells, γδ T cells are a third lineage of adaptive lymphocytes. Less is known about these cells, and they remain relatively untested in cancer immunotherapy. Whilst preclinical evidence supports their utility, the few early-phase trials involving γδ T cells have failed to demonstrate convincing efficacy in solid cancers. Here we review recent progress in our understanding of how these cells are regulated, especially locally within tissues, and the potential for translation. In particular, we focus on the latest advances in the field of butyrophilin (BTN) and BTN-like (BTNL) regulation of γδ T cells and speculate on how these advances may address the limitations of historical approaches in utilising these cells, as well as how they may inform novel approaches in deploying these cells for cancer immunotherapy.

## Introduction

### Beyond the asymptote in cancer immunotherapy: γδ T cells, an untapped resource

Recent advances in our understanding of cancer immunology and the rapid translation of these into clinical applications have transformed the outcomes of many patients with cancer [1]. Given its capacity for specificity as well as memory and self-renewal, nearly all successful cancer immunotherapies to date have leveraged the adaptive immune system. B cell-derived monoclonal antibodies targeting tumour-associated antigens were one of the first immunotherapies to be adopted as the standard of care [2–5]. More recently, αβ T cell-centric immunotherapies have brought about strikingly durable remissions in some patients with otherwise treatment-refractory and/or advanced disease. Immune checkpoint inhibitors (CPIs), which are widely thought to function through de-repression of antigen-specific αβ T cells, have undoubtedly made the most impact to date for solid cancers [6–13]. Other modalities such as adoptive cell therapy (ACT) using chimeric antigen receptor (CAR) bearing T cells and tumour infiltrating T cells (TILs) have also demonstrated promising efficacy, albeit predominantly in haematological malignancies [14–18]. Despite their success and adoption as a standard of care, most patients with cancer do not benefit from CPI therapy. Furthermore, trials of combination CPI therapies, which predominantly target αβ T cells, have resulted in diminishing returns [12, 13, 19, 20], supporting the utilisation of other immune cells with independent modes of action [21, 22].

In addition to B cells and αβ T cells, γδ T cells are a third lineage of adaptive leucocytes that bear antigen receptors generated through somatic recombination. Although the γδ T cell receptor (TCR) was cloned only a few short months after the αβ TCR [23–26], our understanding of these cells and their role in cancer immunology remains limited by comparison. Several factors have contributed to this disparity. Human γδ T cells comprise a small minority of total circulating T cells [27] and even in tissue compartments where they are relatively enriched, they remain a small subset of total T cells [28–31]. Compounding this has been a historical lack of experimental reagents to robustly detect and study these rare cells in clinical samples [32]. Moreover, whilst it is well established that γδ T cells, unlike most αβ T cells, do not require T cell receptor (TCR) engagement with cognate peptide-MHC for activation, our knowledge of γδ TCR ligands remains comparatively incomplete [33]. Furthermore, although murine cancer models have provided mechanistic insight into γδ T cell biology, some murine subsets do not have apparent human counterparts and vice versa [34]. Nonetheless, several conserved and unique properties of γδ T cells have noteworthy implications on cancer immunosurveillance, particularly in solid cancers where achieving durable remission remains a challenge. Unlike αβ T cells, γδ T cells can detect cancers through innate natural killer receptors without the obligate requirement for cognate tumour-associated antigen presentation on MHC [30, 35–37]. These cells are mostly of a memory phenotype with the capacity for rapid functional mobilisation, including the production of tumour-rejecting cytokines and potent cytotoxicity [30, 31, 38–43]. Moreover, many are seeded into steady-state tissues during development, prior to malignancy and without obvious inflammatory triggers [44]. Thus, γδ T cells represent a preformed and local anti-cancer immune surveillance compartment, independent of, but potentially synergistic with, αβ T cells. In support of this, a large pan-cancer study by Gentles and colleagues applied CIBERSORT, an in silico method for determining immune cell composition from bulk gene expression profiles, to microarray data from over 5000 tumours and found a transcriptional signature of γδ T cells to be the strongest predictor of favourable overall survival out of the 22 immune cell subsets detectable [45].

Conscious of the diminishing returns from αβ T cell-centric immunotherapies, notably in solid cancers, here we review the merits of deploying γδ T cells in this setting. In particular, we focus on recent advances in our understanding of the regulation of these cells within tissues and the potential for translation of this into novel therapies for solid cancer.

### γδ T cell subsets

γδ T cell nomenclature remains arcane, even to seasoned immunologists, and thus warrants a brief review here. Similar to αβ T cells, γδ T cells undergo V–(D)–J gene segment rearrangement to generate diverse sets of T cell receptors (TCRs). Like αβ T cells, γδ T cells also comprise distinct functional subsets. However, unlike αβ T cells, which are broadly classified based on CD4 or CD8 expression, γδ T cells are predominantly negative for these co-receptors. The complex ontogeny and classification of these cells is beyond the scope of this review but has been expertly reviewed recently by Mensurado and colleagues [46]. A broad classification of human γδ T cells based on the TCR δ-chain V gene (Vδ) is widely adopted in the field, whilst in mice the cells are commonly classified based on TCR γ-chain V gene (Vγ) use. Of the eight human Vδ genes, Vδ1 and Vδ2 are the most commonly used and thus this review will focus on these subsets. Whilst both Vδ1 and Vδ2 T cells share many similar attributes, such as their capacity for innate-like responsiveness and capacity to kill transformed cells (below), their divergent physiological localisation to peripheral blood (Vδ2 T cells) and body surface tissues (Vδ1 T cells) is noteworthy and discussed. Vδ2 T cells have historically been easier to isolate and expand compared to Vδ1 T cells. Thus, Vδ2 T cells have been better characterised and more frequently utilised in interventional clinical trials (below). On the other hand, whilst Vδ1 T cells have shown promise, they remain relatively untested in the clinic.

## Unique biology of γδ T cells with relevance to solid cancer immunotherapy

### Innate responsiveness independent of cancer (neo)antigens

Current cancer immunotherapies are highly dependent on the presence of neoantigens and/or tumour-associated antigens, reflecting the *modus operandi* of αβ T cells and B cells [47, 48]. Unfortunately, cancers by nature possess a high degree of genomic instability as well as epigenetic plasticity [49]. Suppression of neoantigens [50–53] and/or defects in antigen presentation [54, 55] through cancer-associated genomic instability, epigenetic silencing, or other mechanisms, drive immune evasion and resistance to current immunotherapies. However, neoantigens are not the only route to immunological visibility in cancer. Whilst it may hinder antigen-specific αβ T cell immunosurveillance, genomic instability also drives the expression of immunological stress ligands on cancer cells such as the MIC/ULBP families in humans and the RAE-1/H60/MULT1 families in mice [56, 57]. These molecules are ligands for the natural killer group 2 member D receptor (NKG2D), an innate activating natural killer receptor constitutively expressed by cytotoxic lymphocytes, including innate NK cells, as well as γδ T cells and CD8^+^ αβ T cells. In addition to NKG2D, human γδ T cells can also express numerous other innate activating receptors such as DNAM-1, NKG2C, NKp30 and NKp46 [37, 43, 58–62], the ligands of which are often found on stressed neoplastic cells [63, 64]. The significance of these activating NK receptors was aptly demonstrated in a recent study by Mikulak and colleagues which found a distinct population of Vδ1 T cells expressing numerous NK receptors including NKG2C, NKG2D, NKp30 and NKp46 in human intestinal epithelium [43]. These cells displayed potent NKp46-dependent cytolytic responses against cancer cell lines and their presence within colorectal tumours was strikingly associated with lower-stage disease. More broadly, and in contrast to most αβ T cells, human γδ T cells can be directly activated by these innate receptors, seemingly without the requirement for contemporaneous antigen-specific TCR signalling [30, 37]. Nonetheless, it is also clear these cells can be activated via the γδ TCR, albeit not through classical MHC-peptide engagement but rather through sensing of self-encoded molecules associated with tissue health and distress (discussed below). Once activated, these cells predominantly produce tumour-rejecting cytokines such as IFNγ, release cytotoxic granules and kill target tumour cells [46]. Thus, γδ T cells may provide cancer immunosurveillance via mechanisms independent of antigen-specific adaptive αβ T cells.

### Long-lived tissue residence and cancer immunosurveillance

It is well established that murine tissue-associated γδ T cells are seeded during development into steady-state epithelial tissues without obvious inflammatory triggers, in contrast to αβ T cells which are more commonly recruited later in life following pathogenic challenge and tissue inflammation [65–67]. Genetic deletion of γδ T cells [35, 68], including tissue-specific deletion of signature tissue-resident subsets [36, 69], confers increased susceptibility in de novo murine cancer models. Moreover, this protection from cancer susceptibility was particularly associated with the production of IFNγ from tissue-resident γδ T cells. Interestingly, the phenotype most commonly observed in the absence of tissue-resident γδ T cells was increased numbers of tumours, as opposed to increased size of tumours, suggestive of a critical role for these cells in controlling the early stages of transformation. On the other hand, separate studies have demonstrated a cancer-promoting role for other subsets of murine γδ T cells, particularly those linked to a capacity for IL-17 production [70–73]. Rei and colleagues showed that genetic deletion of total γδ T cells led to reduced tumour size in a transplantable model of ovarian cancer [70]. Subsequent studies have employed antibody-mediated depletion in vivo of γδ T cells and demonstrated protection after depletion of these cells in a breast cancer metastasis model [71], a *Kras*-driven pancreatic cancer model [72] and a *Kras*-driven lung cancer model [73]. These seemingly opposing roles for murine γδ T cells may in part be reconciled by the relatively poor capacity of antibody-mediated depletion strategies to eliminate tissue-resident T cells [74–76]. Hence, studies utilising antibody depletion strategies [71–73] may have disproportionately depleted non-resident, and presumably IL-17-producing γδ T cells, whilst leaving behind IFNγ-producing, tissue-resident γδ T cells. Following this logic, a unifying model supported by both sets of studies is that murine tissue-resident γδ T cells, which are predominantly IFNγ-producing, protect against carcinogenesis whilst non-resident, IL-17-producing γδ T cells may promote it.

Translation of γδ biology from murine to human tissues has been complicated both by technical constraints in studying these rare cells in limited clinical samples as well as by the incomplete conservation of γδ T cells between species. For example, IL-17-producing γδ T cells have proven difficult to find in humans [30, 31, 60, 61, 77]. Nonetheless, several pieces of evidence support a local, tissue-resident γδ T cell compartment with cancer immunosurveillance capacity in humans. γδ T cells have been found in multiple human tissues at steady state including in the skin [78, 79], gut [29, 43, 79, 80], lung [31], breast [30] and liver [81, 82]. Notably, whilst Vδ2 T cells predominate in peripheral blood, it is Vδ1 T cells that appear to be the signature subset within human tissues. Phenotypically, these cells have been shown to express surface molecules important for tissue homing (e.g., CXCR6) [81, 82] and retention (e.g., CD49a, CD103) [30, 31, 43] similar to tissue-resident memory (T_RM_) αβ T cell counterparts [83]. Likewise, tissue-resident Vδ1 T cells have been demonstrated to possess a core T_RM_ transcriptional signature [31] established in human αβ T_RM_ cells [84]. Of note, several studies have demonstrated that these cells express programmed cell death protein 1 (PD-1) [30, 31, 61, 82]. Whilst PD-1 has traditionally been viewed as an inhibitory co-receptor on exhausted T cells, it is becoming increasingly evident that not all PD-1^+^ T cells are terminally exhausted [85]. PD-1 expression in both murine and human T cells appears to be important for survival of these cells within tissues, for their self-renewal and for maintaining their functional competency [86–89]. A recent elegant study by Zakeri and colleagues used donor HLA status to track the persistence of liver-resident γδ T cells in HLA-mismatched recipients after liver transplant [82]. They demonstrated that donor liver-resident γδ T cells, both Vδ1 and Vδ2, can persist for over a decade after transplantation. Compared to non-tissue-resident counterparts, these cells were enriched for PD-1 expression and yet were equally functional as measured by their capacity to produce IFNγ. Separately, PD-1^+^ αβ T cells have been shown to provide the proliferative burst in response to anti-PD-1 treatment which is associated with favourable therapeutic responses in patients with cancer [86, 90] and there is increasing evidence this may also be true for Vδ1 T cells [31, 91]. In summary, human tissues, like murine counterparts, are populated by a bona fide resident population of γδ T cells, particularly of the Vδ1 subset.

Correlative clinical studies across multiple solid cancer types have demonstrated significant associations between the presence of γδ T cells within tumours and clinicopathological features, including tumour size, cancer stage and survival. Whilst some studies have reported an association between intratumoural γδ T cells and adverse features, such as higher-stage disease and/or worse survival, the vast majority have found their presence associated with favourable features (reviewed in [46]). Of note, in studies which have reported on γδ T cell subsets, the presence of Vδ1 T cells has been predominantly associated with favourable features, often independent of other T cell subsets [30, 31, 43, 92]. The presence of γδ T cells in situ at the earliest stages of malignant transformation when antigenic visibility to αβ T cells from accumulated mutations is relatively restricted [93] may underpin their independent and largely favourable prognostic associations [30, 31, 45, 46, 61, 94].

## Clinical translation: challenges and opportunities

The capacity of γδ T cells to recognise and kill transformed cells independent of MHC restriction, their enrichment in barrier tissues from which most solid cancers arise and their association with favourable clinical outcomes (above), has fuelled efforts to develop these cells as “off-the-shelf” cancer immunotherapies. Given their relative ease to isolate and study, nearly all published clinical trials have utilised Vδ2 T cells (recently reviewed in refs. [95, 96] and summarised in Table 1). Of note, most of these trials were conducted in an era where outcomes were dismal for patients with advanced solid cancers and prior to the adoption of CPI therapy as standard of care. Whilst Vδ2 T cell therapies have been shown to be safe and tolerable, objective responses in solid cancers have been virtually absent (Table 1). This across-the-board lack of efficacy is not surprising as nearly all studies have relied on a similar approach to manipulate peripheral blood Vδ2 T cells, albeit with minor variations and in different cancer settings.

**Table 1:** Summary table of published clinical trials of γδ T cells for solid cancer immunotherapy retrieved from PubMed.

Author	Journal	Year	PMID	γδ therapy	Adjunct therapy	Cancer type	Patients treated	Setting	Objective response criteria	Objective responses	Notable findings
Dieli et al. [159]	Cancer Research	2007	PMID: 17671215	Zoledronate +/− low dose IL-2	None/not specified	Prostate cancer	18	Advanced	RECIST	3/18 (0 CR)	Clinical benefit associated with ability of zoledronate to expand and maintain Vδ2 T-cell numbers.
Kobayashi et al. [160]	Cancer Immunology, Immunotherapy	2007	PMID: 16850345	ACT: Vδ2 enriched autologous PBMCs after 2M3B1PP and IL-2 treatment	Low dose IL-2	RCC	7	Advanced	No/not specified	0/7	Two patients demonstrated prolongation of tumour doubling time.
Bennouna et al. [161]	Cancer Immunology, Immunotherapy	2008	PMID: 18301889	ACT: Vδ2 enriched autologous PBMCs after BrHPP and IL-2 treatment	Cycle 1 alone, cycle 2 and 3 with low dose IL-2	RCC	10	Advanced	RECIST	0/10	One dose-limiting toxicity (disseminated intravascular coagulation)
Bennouna et al. [162]	Cancer Immunology, Immunotherapy	2010	PMID: 20563721	BrHPP + low dose IL-2	None/not specified	Mixed solid cancers	28	Advanced	RECIST	0/28	Marked tachyphylaxis with repeated BrHPP
Meraviglia et al. [163]	Clinical and Experimental Immunology	2010	PMID: 20491785	Zoledronate + low dose IL-2	None/not specified	Breast cancer	10	Advanced	RECIST	1/10 (0 CR)	Patients who did not expand Vδ2 T cells had shorter survival than those who did.
Nakajima et al. [164]	European Journal of Cardiothoracic Surgery	2010	PMID: 20137969	ACT: Vδ2 enriched autologous PBMCs after zoledronate and IL-2 treatment	None/not specified	NSCLC	10	Advanced	RECIST	0/10	Elevated plasma IFN-γ post treatment associated with stable disease
Kobayashi et al. [165]	Cancer Immunology, Immunotherapy	2011	PMID: 21519826	ACT: Vδ2 enriched autologous PBMCs after 2M3B1PP and IL-2 treatment	Zoledronate and low dose IL-2	Renal cell cancer	11	Advanced	RECIST	1/11 (1 CR)	Tumour doubling time prolonged in all 11 patients
Lang et al. [166]	Cancer Immunology, Immunotherapy	2011	PMID: 21647691	Zoledronate + IL-2	None/not specified	Renal cell cancer	12	Advanced	RECIST	0/12	Repeated administration of zoledronate and IL-2 associated with reduced proportion of Vδ2 T cells amongst peripheral lymphocytes.
Nicol et al. [167]	British Journal of Cancer	2011	PMID: 21847128	ACT: Vδ2 enriched autologous PBMCs after zoledronate and IL-2 treatment	Zoledronate, 2 patients concurrent chemotherapy, 1 patient concurrent endocrine therapy	Mixed solid cancers	18	Advanced	RECIST	3/18 (1 CR)	Three patients received In-111 labelled Vδ2 T cells and in one patient activity was convincingly seen at adrenal metastasis. All three objective responses also received concurrent endocrine or chemotherapy.
Noguchi et al. [168]	Cytotherapy	2011	PMID: 20831354	ACT: Vδ2 enriched autologous PBMCs after zoledronate and IL-2 treatment	None/not specified	Mixed solid cancers	25	Advanced	No/not specified	0/25	The three patients who experienced a partial response (criteria unclear) also received active concurrent therapy (targeted or chemotherapy).
Sakamoto et al. [169]	Journal of Immunotherapy	2011	PMID: 21304399	ACT: Vδ2 enriched autologous PBMCs after zoledronate and IL-2 treatment	None/not specified	NSCLC	15	Advanced	RECIST	0/15	Updated report of a previous paper by Nakajima et al. 2010
Kunzmann et al. [170]	Journal of Immunotherapy	2012	PMID: 22306909	Zoledronate + low dose IL-2	None/not specified	Renal cell cancer, melanoma	13	Advanced	RECIST	0/13	Well tolerated, one patient with grade 3 fever
Cui et al. [171]	International Journal of Cancer	2013	PMID: 23825037	ACT: Vδ2 enriched autologous PBMCs after zoledronate and IL-2 treatment	Autologous expanded NK cells and αβ T cells at unspecified ratio post RFA	Hepatocellular carcinoma	30 RFA+ACT32 RFA only	Adjuvant	NA	NA	Improvement in PFS in RFA+ACT group compared to RFA alone. Baseline patient characteristics not matched and proportion of γδ T cells infused not specified.
Izumi et al. [172]	Cytotherapy	2013	PMID: 23391461	ACT: Vδ2 enriched autologous PBMCs after zoledronate and IL-2 treatment	None/not specified	Colorectal cancer	6	Advanced/adjuvant	No/not specified	NA	Clinical outcome not reported.
Sugie et al. [173]	Cancer Immunology, Immunotherapy	2013	PMID: 23151944	Zoledronate	None/not specified	Breast cancer	5	Early/advanced	No/not specified	0/5	Repeated administration of zoledronate associated with a reduced proportion of Vδ2 T cells amongst total T cells. No comment on clinical efficacy.
Wada et al. [174]	Cancer Medicine	2014	PMID: 24515916	ACT: Vδ2 enriched autologous PBMCs after zoledronate and IL-2 treatment (intraperitoneal)	Zoledronate (IV and IP)	Gastric cancer	7	Advanced	No/not specified	0/7	Local improvement in reduction of ascites in 2 patients but progression at distant sites.
Pressey et al. [175]	Medicine	2016	PMID: 27684826	Zoledronate + IL-2	None/not specified	Neuroblastoma	4	Advanced	No/not specified	0/4	Well tolerated.
Aoki et al. [176]	Cytotherapy	2017	PMID: 28188072	ACT: Vδ2 enriched autologous PBMCs after zoledronate and IL-2 treatment	Gemcitabine	Pancreatic cancer	28	Adjuvant	No/not specified	NA	No difference in RFS or OS.
Sugie et al. [177]	The Breast	2018	PMID: 29310035	Zoledronate	Letrozole	Breast cancer	55	Neoadjuvant	RECIST	21/55	ORR of the combination was comparable to historical single-agent letrozole response rates.
Alnaggar et al. [178]	Journal for Immuno-Therapy of Cancer	2019	PMID: 30736852	ACT: Vδ2 enriched allogeneic PBMCs after treatment zoledronate + undisclosed cytokines	None/not specified	Cholangiocarcinoma	1	Advanced	No/not specified	0/1	Well tolerated, no objective response criteria.
Kakimi et al. [179]	Journal for Immuno-Therapy of Cancer	2020	PMID: 32948652	ACT: Vδ2 enriched autologous PBMCs after zoledronate and IL-2 treatment	None/not specified	NSCLC	25	Advanced	RECIST	0/25	One patient demonstrated response in lung lesion but progression with new liver metastases.
Lin et al. [180]	Signal Transduction and Targeted Therapy	2020	PMID: 33093457	ACT: Vδ2 enriched allogeneic PBMCs after zoledronate and IL-2 treatment	None/not specified	Pancreatic cancer	30 Vd2+IRE32 IRE only	Advanced	No/not specified	0/30	Modest improvement in survival in Vδ2+IRE arm
Gassart et al. [181]	Science Translational Medicine	2021	PMID: 34669444	Anti-BTN3A agonist antibody	None/not specified	Mixed solid cancers	6	Advanced	No/not specified	0/6	Reduction in circulating Vδ2 T cells after treatment potentially reflecting recruitment to the tumour bed.
Xu et al. [182]	Cellular and Molecular Immunology	2021	PMID: 32939032	ACT: Vδ2 enriched allogeneic PBMCs after zoledronate, IL-2, IL-15 and vitamin C treatment	IRE, Iodine-125 and/or cryoablation	Lung and liver cancer	132	Advanced	RECIST	1/132 (1 CR)	One case of complete response in patient who also had concurrent iodine-125 therapy.

*2M3B1PP* 2-methyl-3-butenyl-1-pyrophosphate, *ACT* adoptive cell therapy, *BrHPP* bromohydrin pyrophosphate, *CR* complete response, *IL-2* interleukin-2, *IP* intraperitoneal, *IRE* irreversible electroporation, *IV* intravenous, *NSCLC* non-small cell lung cancer, *PBMC* peripheral blood mononuclear cell, *PFS* progression-free survival, *ORR* objective response rate, *OS* overall survival, *RCC* renal cell carcinoma, *RECIST* Response Evaluation Criteria in Solid Tumours, *RFA* radiofrequency ablation.

### Vδ2 T cells: off the beaten path

It has been known for some time that Vδ2 T cells, specifically Vγ9Vδ2 T cells, can be activated by phosphorylated intermediates of cholesterol metabolism known as phosphoantigens (pAgs) [97–100]. Dysregulated cholesterol metabolism and subsequent accumulation of pAgs in cancer cells contributes to the near-universal cytotoxicity displayed by Vδ2 T cells in vitro against cancer cell lines [95, 101]. Thus, attempts to harness Vδ2 T cells in the clinic have largely involved activating and expanding these cells through the provision of pAg, either directly or indirectly through treatment with bisphosphonates which increase pAg accumulation [96]. Most trials have either attempted activation in vivo of Vδ2 T cells through systemic delivery of pAgs/bisphosphonates or activation ex vivo of peripheral blood-derived Vδ2 T cells using pAgs/bisphosphonates followed by ACT. Despite the cells’ unequivocal cancer-killing capacity in vitro, their clinical efficacy has been disappointing (Table 1). Several plausible explanations have been proposed for this conspicuous discrepancy. The systemic utility of bisphosphonates is likely hindered by their unfavourable pharmacokinetic profiles for tumour immunotherapy. These drugs are rapidly cleared from the circulation through renal excretion and bone absorption with very little delivery to the soft tissues [102, 103]. Hence, the extent to which systemic bisphosphonates activate Vδ2 T cells within the tumour bed remains largely unclear. Likewise, there is little evidence to suggest that Vδ2 T cells activated ex vivo can traffic to tumours or be retained within them [104]. In fact, pAg activation of Vδ2 T cells induces expression of lymph node homing chemokine receptors [105], probably related to their capacity for professional antigen presentation [106–108]. Moreover, global activation of Vδ2 T cells by systemic pAgs has been linked to the exhaustion/anergy of these cells in preclinical primate models as well as in clinical trials [109–111].

**Fig. 1:**
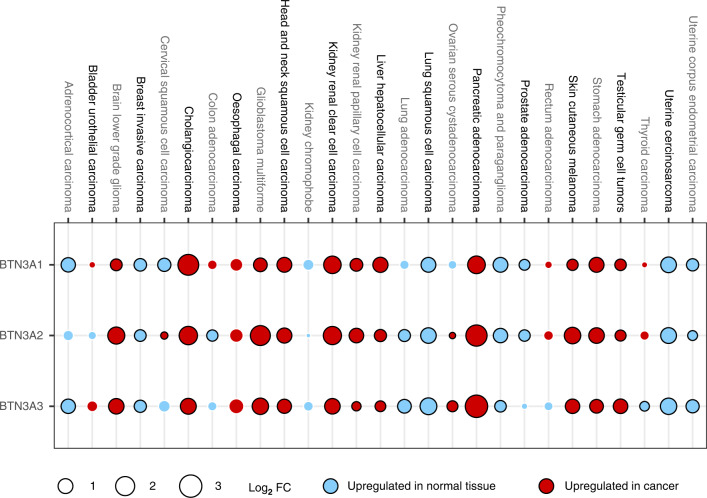
Expression of BTN3A isoforms in normal tissues and solid cancers. Gene expression profiles of each BTN3A isoform were extracted from the OncoDB database (https://oncodb.org/cgi-bin/genomic_normal_expression_search.cgi) and plotted as log_2_ fold change between median expression levels in each cancer and corresponding normal tissue. Red fill denotes increased expression in a specific cancer type compared with corresponding normal tissue. Blue fill denotes increased expression in normal tissue compared with corresponding cancer. Size of circles are proportional to log_2_ fold change. Black border denotes unadjusted *P* value < 0.05.

Although phosphoantigens have long been known to activate Vδ2 T cells [100], the mechanism by which they do so has only recently been described. Seminal work by Harly and colleagues first established butyrophilin 3A1 (BTN3A1) to be critical for Vδ2 phosphoantigen reactivity [112]. Subsequent work has implicated other members of the butyrophilin family, including BTN2A1, BTN3A2 and BTN3A3 [113–115]. Butyrophilin (BTN) and related butyrophilin-like (BTNL) molecules (below) are human immunoglobulin (Ig) superfamily receptor proteins considered to be part of the wider B7 family of receptors [116]. Other B7 family members include important immunomodulatory receptors such as CD80 (B7.1), CD86 (B7.2) and PD-L1 [116]. Accordingly, BTN molecules have been reported to be immunosuppressive for αβ T cells [117, 118]. On the other hand, these molecules have now also been shown to be important for the phosphoantigen-dependent activation of Vδ2 T cells. Current evidence suggests that BTN2A1 is required for TCR binding via the γ-chain of Vγ9Vδ2 T cells [114, 115] whilst the intracellular domain of BTN3A1 is required for phosphoantigen sensing [119], and both BTN2A1 and BTN3A1 are required for pAg reactivity. Moreover, BTN3A2 and BTN3A3 appear to be important for the optimal function of BTN3A1 through the regulation of its subcellular trafficking [113]. Whilst BTN molecules have been found to be expressed by many cell types, their expression has been particularly associated with epithelial tissues, albeit without any obvious tissue bias [116]. However, what is striking is their differential expression in steady-state epithelial tissues versus their respective neoplastic counterparts. This is most evident in BTN3A isoforms which are often upregulated in cancers relative to normal tissue counterparts [118, 120–123] (Fig. 1).

These recent advances in our understanding of the molecular determinants of Vδ2 T cell activation have clear potential for translation. Where historical attempts to harness Vδ2 T cells through blunt application of pAgs/bisphosphonates have proven largely ineffective, targeted modulation of tissue-associated regulators, such as BTN molecules (discussed below), may overcome barriers like poor tumour homing and/or pAg-associated anergy (discussed above). Moreover, cancer-associated upregulation of BTN3A isoforms (above) relative to normal tissues may provide an important therapeutic window. One promising approach involves the use of agonistic BTN3A antibodies which induce conformational changes mimicking those caused by pAg [112, 124]. Compared with bisphosphonates, antibodies have considerably longer plasma half-lives and thus offer greater cumulative tumour penetration. Indeed, a recent Phase I study of ICT01 (EVICTION, NCT04243499), an agonistic pan-BTN3A antibody, found a marked drop in circulating Vδ2 T cells shortly after ICT01 dosing in all patients. Whilst several potential explanations may underpin this observation, including activation-induced cell death or antibody-dependent depletion, the authors propose the loss of circulating Vδ2 T cells reflects their recruitment to tumours, presumably as a consequence of ICT01-dependent BTN3A agonism on tumour cells. In support of the latter, they found clear evidence of increased Vγ9^+^ γδ T cells in the tumour bed after ICT01 treatment in one patient where paired pre/post treatment tumour biopsies were evaluable. Thus, treatment with this agonistic BTN3A antibody may improve recruitment and retention of Vδ2 T cells within the tumour microenvironment. ICT01 was well tolerated with no dose-limiting toxicities in this study. Indeed, in vitro assays showed that treatment preferentially increased killing of cancer cells compared with non-malignant cells by PBMCs, potentially reflecting the overexpression of BTN3A isoforms in cancers versus normal tissues. Furthermore, the authors did not observe any evidence of ICT01-induced exhaustion of Vδ2 T cells in vitro over the course of several days’ exposure, a common Achilles’ heel of the bisphosphonate/pAg approach. Given their homology with other B7 family receptors, including PD-L1, BTN3A isoforms have also been reported to be suppressive for αβ T cells [117, 118]. In a case of two birds with one stone, Payne and colleagues demonstrated that agonistic BTN3A antibodies not only activate Vδ2 T cells but also relieve BTN3A1 suppression of αβ T cells. The authors further explored the impact of agonistic BTN3A antibodies using immunodeficient mice reconstituted with human γδ T cells and/or human αβ T cells bearing a chimeric antigen receptor (CAR) against a transplanted ovarian cancer cell line. They found that maximal protection was achieved through co-administration of γδ T cells, αβ CAR T cells and agonistic BTN3A antibody, suggesting that these antibodies may enable concerted anti-tumour responses by these cells. Importantly, they also demonstrated significantly improved recruitment of Vγ9^+^ γδ T cells into tumours after agonistic BTN3A antibody treatment [118]. It remains to be seen if such approaches targeting tissue intrinsic regulatory axes of Vδ2 T cells may help to break the duck in a string of disappointing trials of these cells. Nonetheless, the early evidence has been promising and the community eagerly awaits the results of ongoing Phase 2 efficacy trials.

### Vδ1 T cells: the road less travelled

Whilst novel approaches may breathe new life into Vδ2 T cell-based cancer immunotherapy, the major subset of γδ T cells within human tissues are Vδ1 T cells and these cells remain relatively untested in this context. Recent advances in the capacity to isolate and study Vδ1 T cells coupled with a growing interest in cancer immunosurveillance by tissue-resident T cells [125, 126] has rapidly accelerated our understanding of these cells’ biology. Vδ1 T cells possess multiple qualities which support their utilisation for cancer immunotherapy. Like Vδ2 T cells, Vδ1 T cells can both recognise and kill transformed cells innately via activating NK receptors [30, 37, 43, 60]. Nonetheless, these cells possess several potential advantages. For starters, they can express diverse activating natural cytotoxicity receptors (e.g., NKp30, NKp46) not commonly found on Vδ2 T cells [37, 43, 60]. Unsurprisingly, given their association with epithelium, Vδ1 T cells also more commonly express receptors for tissue homing and retention [30, 31, 91]. Thus, when considering adoptive cell therapy for solid cancers where tumour bed penetration is often considered a barrier [127], Vδ1 T cells may have an edge over cells derived from the systemic circulation. The indigenous nature of Vδ1 T cells within steady-state tissues and their presence in tumours raises the appealing possibility for therapeutic manipulation of these cells in situ using monoclonal antibodies or other cell engagers. Compared with adoptive cell therapy, cell engagers can be more readily engineered to target different ligands and are easier to administer as well as considerably cheaper to produce. In a recent study of patients with NSCLC, the presence of tissue-resident Vδ1 T cells in non-tumour adjacent lung tissue was highly predictive of disease-free survival after surgery, consistent with an immunosurveillance role for these cells [31]. Hence, an understanding of how these Vδ1 T cells are regulated within tissues can identify key therapeutic targets to maximise their utility in situ.

That Vδ1 T cells reside within barrier tissues at steady state raises the intriguing prospect that these cells possess intrinsic tissue-specific adaptations particularly suited to cancer immunotherapy applications. Specifically, it seems conceivable that these cells would have the ability to detect normality, thus avoiding spurious activation, and yet at the same time are primed for rapid and innate effector functions in response to the inevitable and disparate challenges imposed by the external environment on barrier tissues. Formally establishing this proposed dichotomy and an appreciation of the underlying local, tissue-centric cellular and molecular regulators could unlock the holy grail of cancer immunotherapy, namely tumour rejection without tissue toxicity. Combined with insights from murine models, recent studies have begun to resolve how Vδ1 T cells are regulated within human tissues and cancers, and provide some early evidence for this hypothesised functional dichotomy. One key and conserved regulatory axis is that of butyrophilin-like (Btnl/BTNL) molecules and tissue-resident γδ T cells in barrier tissues [29, 128]. Within the murine gut, epithelium-specific expression of Btnl1, Btnl4 and Btnl6 have been shown to regulate the development, tissue retention and maintenance of intraepithelial Vγ7^+^ γδ T cells. [29, 129]. Likewise, human gut-specific expression of BTNL3 and BTNL8 likely regulates Vγ4^+^ (frequently paired to Vδ1) intraepithelial lymphocytes (IELs) [29, 128, 130, 131].

**Fig. 2:**
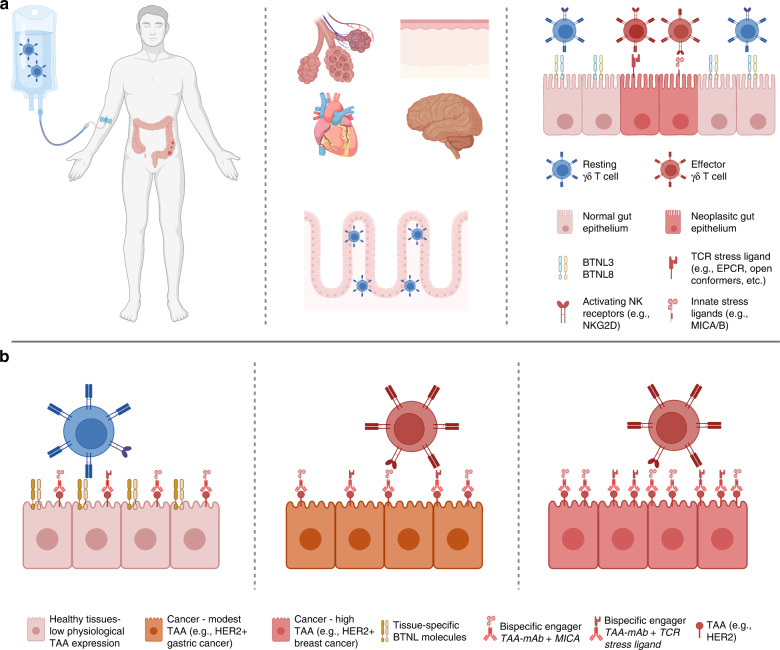
Translating tissue biology of V**δ**1 to effective cancer immunotherapy. **a** Adoptively transferred, tissue-derived Vδ1 T cells (left panel) may preferentially traffic to and accumulate in target organs dependent on tissue-specific BTNL expression (middle panel). This could reduce detrimental activation in uninvolved organs and thus improve therapeutic windows (middle panel). Within target organs, the hypothetical logic-gate functionality of the TCR may provide further fine-tuning of Vδ1 T-cell activation to target neoplastic cells whilst sparing healthy cells. **b** Clinically relevant TAAs, such as human epidermal growth factor receptor 2 (HER2), which are expressed at low levels on most healthy epithelial cells and only modestly upregulated on cancer cells (e.g., gastric cancer, treatment-resistant HER2^+^ breast cancers) can be difficult to target with tolerable safety windows using a “single argument” approach (e.g., a monoclonal antibody). The hypothetical logic-gate functionality of the γδ TCR to permit activation based on both the absence of normality and the presence of stress can be exploited for increased therapeutic windows. Bispecific engagers, which have excellent tissue penetration, can recognise TAAs via a monoclonal antibody (mAb) domain whilst engaging γδ T cells via a TCR stress ligand. Binding of bispecific engagers to physiologically expressed TAAs on healthy cells would not be sufficient to trigger γδ T-cell activation as these cells still express normality-associated tissue-specific BTNL molecules (left panel). On the other hand, the downregulation of BTNL molecules on neoplastic cells in combination with bispecific engagement permits activation of γδ T cells within tumours (middle and left panel). Moreover, the modular nature of bispecific antibodies allows for bespoke tuning by targeting of other activating axes on γδ T cells (e.g., innate NK receptors).

Notably, Btnl/BTNL expression also appears to be linked to tissue health, evoking the differential expression of BTN3A isoforms in steady-state versus neoplastic tissues (above). However, unlike BTN3A isoforms which appear to be upregulated in cancer, expression of Btnl/BTNL is most often lost in dysregulated tissue states including inflammation and cancer, compared to steady-state [131, 132]. It is therefore tempting to speculate that Btnl/BTNL molecules may signal “normality” to tissue-resident Vδ1 T cells and potentially restrain pernicious activation within healthy tissues. Indeed, this hypothetical model was proposed recently by Hayday and Vantourout [133]. Specifically, they proposed that tissue-specific Btnl/BTNL molecules expressed at steady state bind to an “innate” germline-encoded region on the TCR γ chain of tissue-resident γδ T cells, and that this interaction supports the maintenance of signature Vγ subsets of these cells within tissues (e.g., BTNL3/8 and Vγ4^+^ IEL in the human gut) but also prevents the engagement of the γδ TCR (incorporating both γ chain and δ chain) with cognate, self-encoded, complementarity-determining region 3 (CDR3)-dependent ligands induced upon tissue stress. Thus, in settings of tissue dysregulation, such as cancer, where BTNL expression is often downregulated [132, 134–136], resident γδ T cells may then be released to respond to putative CDR3-dependent, stress-induced activating ligands. In support of this model, a Vγ4^+^ γδ TCR with defined clonal CDR3 reactivity [137] has recently been demonstrated to recognise both BTNL3 via a germline-encoded region of the γ chain, as well as the endothelial protein C receptor (EPCR) via the CDR3 [128, 130, 137]. BTNL3 is expressed at steady state by the intestinal epithelium but is markedly downregulated in colon cancer [29, 132] whilst EPCR, a stress-induced MHC class I-like molecule, is frequently overexpressed in multiple cancers including colon cancer [138–140]. Importantly, BTNL3 was shown to have a higher affinity for the TCR compared to EPCR (*K*_d_ ~ 15–25 μM versus *K*_d_ ~ 90 μM) and could inhibit EPCR binding [130, 137]. Indeed, multiple CDR3-dependent Vδ1 TCR ligands have now been identified and several are also MHC class I-like molecules [33, 141–144]. Intriguingly, a recent study of Vδ1 T cells derived from mismatch repair deficient colorectal cancers found that these cells displayed enhanced reactivity towards patient-derived tumour organoids engineered to be deficient for β2m compared with parental organoids [91]. The authors speculated that lower MHC class I expression in β2m-deficient organoids may activate Vδ1 T cells via reduced inhibitory killer cell immunoglobulin-like receptor(s) engagement. An alternative and provocative explanation would be that these Vδ1 T cells recognised “open conformers” of MHC class I, i.e., heavy chains in the absence of β2m and peptide, as has been reported in the context of CMV infection by Dechanet-Merville and colleagues [145]. Thus, a picture emerges in which the TCRs of tissue-resident Vδ1 T cells effectively act as logic-gates that permit the cells’ activation based firstly on the absence of normality and secondly on the presence of tissue stress. This firmly remains a model at present and indeed the mechanisms by which the γδ TCR may distinguish between engagement of germline-encoded versus CDR3-encoded regions remain unclear and are an active area of research. Nonetheless, the potential implications on Vδ1-based cancer immunotherapies may be profound. In particular, the hierarchical precedence of normality over stress could enable large therapeutic windows for Vδ1-based immunotherapies. For example, adoptively transferred Vδ1 T cells derived from a colorectal cancer may traffic back to the colon but would only become activated within the tumour microenvironment where BTNL3 and BTNL8 loss (absence of normality) is concurrent with upregulation of CDR3-dependent TCR stress ligands (e.g., EPCR, open conformers etc.) and/or other activating ligands (e.g., MICA, ULBPs, etc.) (Fig. 2a). Likewise, bispecific cell engagers could target clinically relevant tumour-associated antigens that are also expressed at low levels in normal tissues (e.g., HER2) whilst potentially avoiding on-target, off-tumour toxicity (Fig. 2b). Looking beyond the hypothetical, several groups have now demonstrated the capacity to generate large numbers of Vδ1 T cells in vitro [30, 60, 146] and a first in human trial of these cells for cancer immunotherapy is currently underway (NCT05001451). Thus, our growing understanding of the basic biology of these cells may have rapid and proximal clinical implications.

### γδ T cells in the era of CPI therapy

Although CPI therapies benefit only a minority of patients, this is still a considerable minority. Moreover, CPIs have become gold-standard first-line treatments with curative potential for many cancer types, even in the metastatic setting [13, 147–150]. Thus, the clinical landscapes within which contemporary and future trials of γδ T cell therapies must now operate have become vastly more competitive compared to historical studies (Table 1). Of the many CPIs currently available, anti-PD-(L)1 therapies have consistently proven to be the most efficacious [8, 13, 151] and now comprise the backbone of a large proportion of immunotherapy combination trials, including those involving novel agents [152]. However, these immune checkpoint inhibitors have largely been studied in the context of αβ T cells. Whilst both Vδ1 and Vδ2 T cells can express PD-1 [30, 60, 61, 91], whether or not γδ T cells are regulated by these checkpoints is less clear [30, 91, 153–155] and merits urgent attention. Germane to this, an intratumoural transcriptomic signature of Vδ1 T cells has recently been shown to be predictive of response to anti-PD-1 therapy in a cohort of patients with mixed solid cancers [31]. Moreover, in mismatch repair deficient colorectal cancers with β2m loss, treatment with anti-PD-1 was associated with an increase in intratumoural Vδ1 T cells [91]. Thus, in a fortuitous moment of scientific serendipity, the inevitable combination trials of γδ T cells and anti-PD-(L)1 therapies (see above) may turn out to be an entirely rational combination.

## Concluding remarks

Most contemporary combination immunotherapies work by modulating αβ T cells, often through targeting multiple inhibitory and/or activating checkpoints. Whilst this approach has yielded some remarkable successes [12, 13], recent combination trials have demonstrated only incremental gains in efficacy [20]. Moreover, meta-analyses of clinical trials have provided compelling evidence that combination therapies are most effective when individual mechanisms of action are independent [21, 22]. In this respect, γδ T cells represent ideal therapeutic targets given their distinct yet complementary role in cancer immunosurveillance alongside αβ T cells. A renewed and growing appreciation of the potential of γδ T cells is reflected in several recent and comprehensive reviews of their utility in cancer immunotherapy [46, 156, 157]. To these reviews, we add here a more speculative perspective on the potential translation of recent discoveries in the basic immunobiology of these cells to effective clinical therapies.

## Data Availability

Not applicable.
